# Statistical Evaluation of the Material-Source Effects on the DSR Rheological Properties of Plant-Mix Extracted Asphalt-Binders

**DOI:** 10.3390/ma14081931

**Published:** 2021-04-13

**Authors:** Lubinda F. Walubita, Jose G. Gonzalez-Hernandez, Gilberto Martinez-Arguelles, Hossain Tanvir, Luis Fuentes, Seyed Amid Tahami

**Affiliations:** 1 Texas A&M Transportation Institute (TTI), The Texas A&M University System, College Station, TX 77843, USA; L-Walubita@tti.tamu.edu; 2 Department of Civil & Environmental Engineering, Universidad del Norte (UniNorte), Barranquilla 081001, Colombia; jgonzalezg@uninorte.edu.co (J.G.G.-H.); lfuentes@uninorte.edu.co (L.F.); 3 Central Structural Discipline, Subsea 7, Houston, TX 77094, USA; tanvir_ha@hotmail.com; 4 Department of Civil and Environmental Engineering, University of Texas at San Antonio, San Antonio, TX 78249, USA; seyedamid.tahami@utsa.edu

**Keywords:** Texas DSS, asphalt-binder, DSR, rheology properties, material-source effect, statistical analysis

## Abstract

The work presented in this paper was carried out to statistically evaluate and quantify the material-source effect on the asphalt-binder’s rheological properties using Analysis of Variance (ANOVA) and Tukey’s Honestly Significant Difference (Tukey´s HSD) test. The study focused on the Asphalt-Binders’ high-temperature rheological properties, namely, the G*, δ, G*/Sin(δ) and G*/(1 − (1/Tan(δ)Sin(δ))) parameters, measured using the Dynamic Shear Rheometer (DSR) device. The DSR data analyzed in the study were extracted from the Texas flexible pavements and overlays database, namely, the Texas Data Storage System (DSS), covering two Asphalt-Binders (ABs), performance grade (PG) 64-22 and PG 76-22 plant-mix extracted ABs that were treated as rolling thin film oven (RTFO) residue, and sourced from 14 different suppliers. The study findings substantiate that material-source has an effect on the high-temperature rheological properties of ABs. Additionally, it was also concluded that in as much as performance superiority and costs are crucial issues in deciding the AB source/provider, consistency and quality aspects cannot be disregarded. Therefore, material-source effects should be inclusively evaluated from both performance (rheological properties) and quality (consistence) standpoints as well as cost considerations when choosing a supplier. In general, the study contributes to the state-of-the-art enrichment on aspects of material-source effects on RTFO residue ABs’ high-temperature rheological properties, consistency, variability, and data quality.

## 1. Introduction

The asphalt-binders (ABs) used in the design of hot-mix asphalt (HMA) is one of the determining factors in its performance throughout its service life. ABs are commonly produced from the processing of crude oil by applying atmospheric or vacuum distillation including mild oxidation (often referred to as air rectification or semi-blowing) processes. Mildly oxidized ABs, which are often used to produce semi-blown (air-rectified) paving ABs, have physical properties that are like those of atmospheric or vacuum-distilled paving ABs [1]. In general, ABs used to produce HMA mixes are often classified based on their physical and visco-elastic properties such as the viscosity and stiffness (i.e., complex shear modulus) [2,3,4,5,6]. However, some source-dependent chemical properties, such as the asphaltenes, resins, and oils, have a profound impact on the ultimate visco-elastic and physical properties of the AB. Asphaltenes, for instance, influence the strength and stiffness, resins are related to adhesion, and the oils are directly related to viscosity and fluidity [1,7].

Among others, the pavement industry depends on the physical properties to characterize and quantify the performance of HMA, which are partly driven by the chemical and rheological properties of the ABs. The Superpave and its performance grading (PG) system are predominantly based on the physical properties of ABs measured in the laboratory with consideration of the prevailing climatic and environmental conditions such as temperature [2]. Additionally, the Superpave system also considers the short- and long-term aging of the AB that normally takes place during HMA production, placement operations, and the service life of asphalt pavements, respectively [8].

Current AB production processes have been considerably enhanced, among others, due to economic, technical, and environmental evolutions [9]. Crude oil sources and product demands have substantially evolved, and, as a result, refining technologies have had to innovatively adapt, including the AB suppliers [9]. This has partially resulted in an increasing AB variability and quality inconsistency, which may not be adequately considered in the current specifications [9]. Problematic ABs often occur when the blends are not well controlled or formulated. The ultimate consequences are pavement issues in terms of lower durability and reduced longevity such as premature rutting or cracking [10].

Some studies have highlighted that the ABs’ rheological properties have a significant influence on the HMA properties and performance [11,12,13]. Similarly, other studies (mostly on original unaged ABs) have also shown that material source has an influence on both the ABs’ rheological properties and the overall HMA performance [12,14,15]. For the work presented in this paper, a statistical analysis was carried out to assess and quantify the influence of material-source on the high-temperature rheological properties of ABs measured using the dynamic shear rheometer (DSR) test device [16]. Different from most of the literature, this study specifically focused on ABs that were recovered from plant-mix materials and treated as rolling thin film oven (RTFO) residue. In addition to focusing on short-term aged ABs (namely, RTFO residues recovered from plant-mix materials), another novelty of the study is that it comparatively evaluated different AB high-temperature rheological properties, including the complex shear modulus (G*), phase angle (δ), G*/Sin(δ), and G*/(1 − 1/Tan(δ)Sin(δ))) parameters from in-service road sections.

In this study, ANOVA and Tukey’s HSD statistical methods were applied to comparatively evaluate up to 14 different sources/suppliers of Abs, involving PG 64-22 and PG 76-22 Abs, all recovered from plant-mix materials that were hauled from field construction jobsites. In the following subsections of the document, a critical review of the previous research effort is provided followed by the study matrix plan, test results, statistical analyses, and discussion of the study findings. The study then concludes with a summary of key findings and conclusions.

## 2. Literature Review

Given the enhancements in the production of ABs over the years, due, among other reasons, to economic, environmental, technical refining, and even geopolitical factors, that have a direct influence on the ABs and ultimately on the physical properties and performance of HMA mixes, the asphalt industry has faced new challenges in terms of material quality control, consistency, and premature failures in the field [15]. On the other hand, state road agencies as well as the transportation and construction industries keep demanding better quality and superior ABs so as to optimize their HMA mix-designs and maximize pavement performance [10]. As a result, research studies oriented towards verifying and improving the chemical, physical, and rheological properties of ABs have been scaled up and are now more critical than ever, particularly with the ever dynamically changing traffic loading regime and environmental conditions [1,8,9,10].

Many studies (mostly on original unaged ABs) have been carried out on the variability and differences in the rheological properties of different ABs in relation to their ultimate effects on the properties and behavior of HMA mixes [11,13,15,17]. However, based on the literature reviewed, there are limited efforts on the influence of the sources and providers on the rheological properties of ABs, particularly on plant-mix recovered ABs and RTFO residues (i.e., AB subjected to short-term aging). Planche et al. [10] conducted a study focused on fingerprinting ABs to evaluate the source variability and its impacts on the AB’s chemical composition and rheological properties. Their findings indicated a huge variation in the chemical composition among the ABs from different sources, even for those with the same PG classification. As theoretically expected, these variations in the chemical compositions and rheological properties, including the degree of aging of the ABs, ultimately had an impactful effect on the HMA mix stiffness (modulus), rutting resistance, cracking, and fatigue damage [10]. Lill et al. [14] studied a total of 15 ABs using the Superpave performance characteristics including the AB stiffness. Their findings indicated that the stiffness and high-temperature performance grades of the ABs varied among the suppliers as they were sampled from different geographical areas and crude oil sources. Differences of up to 10 °C in the performance grades were observed among supposedly similar AB types that came from different suppliers and sources [14].

Alvarez et al. [12] studied the variability of 18 ABs classified as Pen 60-70 characterized using both the traditional indices and fundamental material properties including rheological, thermodynamic, and chemical properties. The results showed that while the ABs presented values of the coefficient of variation (CoV) less than 10% for the penetration and softening point indices, advanced testing such as the rheology, surface free energy (SFE), and chemical composition analysis presented high variability, with CoV values exceeding 20% among the different AB sources.

Evidently, the above literature review [10,12,14], mostly based on original unaged ABs, provides insights on the fact that ABs (even those with the same PG grade) from diverse providers and sources could undesirably exhibit variations in terms of the chemical composition and rheological properties that are directly related to performance. To further add to this knowledge and enhance the literature, particularly with respect to short-term aged RTFO residues, this study applied statistical methods to quantitatively assess the material-source effects on the AB’s rheological properties. Specifically, the study focused on the high-temperature rheological properties of the plant-mix recovered “RTFO residue” ABs measured using the DSR test device and involved two commonly used Texas ABs, namely, PG 64-22 and PG 76-22 from 14 different suppliers. All the ABs (RTFO residues) were extracted from plant-mix materials that were directly hauled from field construction sites.

### 2.1. Asphalt-Binder High-Temperature Rheological Properties

Based on the Superpave specification, the high-temperature rheological properties of the ABs are commonly measured, characterized, and quantified in the terms of the rutting parameter, G*/Sin(δ), using the DSR test device—where the numerator (G*) represents the complex shear modulus of the AB and the denominator is the Sine of the phase angle (δ) [16,18]. However, due to some inadequacies reported about the G*/Sin(δ) parameter, particularly with respect to polymer modified ABs and their poor correlation-ship with HMA field rutting performance [19,20], Shenoy [21] proposed the G*/(1 − (1/Tan(δ)Sin(δ))) parameter (for δ ≥ 55°) as a supplement to better characterize and quantify the high-temperature non-recoverable response of the ABs. Theoretically, it is assumed that Tan(δ) captures the rutting resistance potential of ABs better than Sin(δ) due to its characteristic nature to quantitatively approach 1.0 at high temperatures [22]. Note that for RTFO aged AB residues, 2.2 kPa and 55°, respectively, are often used as the Superpave screening and temperature grading criteria [16,18,19,21,22,23].

Other parameters often used to characterize the AB high-temperature rheological properties include the elastic recovery (R) and non-recovery creep compliance (Jnr) measured from the elaborative multiple stress creep-recovery (MSCR) test protocol, also using the DSR test device [19]. While the MSCR test protocol (R and Jnr) is reported to provide better quantification of the elastic response-behavior and presence of polymer modifiers in ABs (but with more complex computation/analysis procedures [20]), this study’s focus was on the more commonly used G*, δ, G*/Sin(δ), and G*/1 − (1/Tan(δ)Sin(δ) parameters relative to material-source effects [19,23]. Compared to R and Jnr, these parameters (G*, δ, G*/Sin(δ), and G*/(1 − (1/Tan(δ)Sin(δ))) are also much easier to measure and compute/analyze [18].

### 2.2. Recycled Asphalt Pavement (RAP), Recycled Asphalt Shingles (RAS), and Plant-Mix Recovered Asphalt-Binders

With ABs recovered from plant-mix materials (i.e., RTFO residues), as was the case in this study, HMA mix additivities such as RAP, RAS, etc., usually tend to increase the proportion of the aged AB in the total AB blend [24,25,26]. Thus, in addition to increasing the stiffness of the AB blend and ultimately that of the resulting HMA mix including rutting resistance improvements, these additives (RAP/RAS) also have the potential to impact the consistency and variability of the rheological properties of the plant-mix extracted ABs. However, comprehensive study of the RAP/RAS effects was outside the scope of this paper as the study’s focus was on the material-source effects.

## 3. Study Matrix Plan

The experimental design and matrix plan consist of using the Texas database for flexible pavements and overlays, denoted as the Texas Data Storage System (DSS), as the primary data source for the study [18]. The database is discussed in this section of the paper along with the laboratory test, the ABs, and the statistical methods used to analyze the data.

### 3.1. Asphalt-Binder Data Source (the Texas DSS)

The Texas DSS is an Microsoft (MS) Access comprising over 100 Texas flexible pavements and overlays [18,27,28] highway sections with extensive laboratory and field performance data that include design, as-built cross-sectional drawings, construction, layer material properties (both laboratory and field measured), traffic, climate, overlays’ existing distresses, and field performance data—which have been periodically collected since 2010.

The extensive material properties in the Texas DSS include the laboratory measured AB rheological properties from the DSR test device—which are the subject of this paper [18,29]. In addition to the processed and analyzed data, the DSS has an accompanying raw data storage system that contains all the corresponding raw data/files, such as the DSR test data files. Full details of the DSS and RDSS can be found in Walubita et al. [18,28,29].

### 3.2. Laboratory Measurement of the Rheological Properties (the DSR Device)

As per the Texas DSS protocol, laboratory testing with the DSR device [16,23] was conducted on the ABs extracted from plant-mix materials that were directly hauled from the field construction sites and treated as RTFO residues [18,28]. A centrifugal extraction method with a chlorinated solvent was used for extracting the ABs from the pre-heated loose HMA (plant-mix) that were hauled directly from the field construction jobsites [27]. Using the DSR device [16,23], testing was conducted on the extracted ABs at an oscillating shear-frequency of 1.59 Hz (corresponding to 90 km/h) in a sinusoidal shear-strain waveform following the test procedures specified in AASHTO T240 and T315, respectively [23,30]. A minimum of three specimen replicates, 25 mm in diameter by 2 mm thick, were fabricated and tested per AB at multiple temperatures ranging from 58 to 82 °C [18,27,29].

Using the DSR test device [16,23], the high-temperature rheological properties of the ABs were measured and quantified in terms of the complex shear modulus (G*) and phase angle (δ)—where δ is the time lag between the maximum applied stress (τ_max_) and the maximum shear strain (γ_max_). The ratio of τ_max_ and γ_max_ is G*. G* represents the specimen’s total resistance to deformation when repeatedly sheared, while δ quantifies the visco-elastic behavior of the AB, namely, the higher the δ value in magnitude, the more viscous the AB is and vice versa [30]. In line with the Texas DSS protocol, the entire data output from laboratory testing with the DSR device [18,27], namely, the G*, δ, and G*/Sin(δ), is all catalogued and can be found in the DSS. Additionally, the G*/1 − (1/Tan(δ)Sin(δ) parameter was also computed and analyzed in this paper [21]. As previously mentioned, 2.2 kPa and 55°, respectively, are often used as the Superpave screening and temperature grading criteria for RTFO aged AB residues [16,18,19,21,22,23].

### 3.3. Material Sources and Asphalt-Binders (Plant-Mix Extracted)

As extracted from the Texas DSS, 14 sources and suppliers of ABs, covering PG 64-22 and PG 76-22 RTFO residues, were statistically evaluated [18,27]. The ABs, from different suppliers were denoted as “Source01 through to Source14”, for impartial anonymity, are listed in Table 1.

Note that with the plant-mix extracted ABs (i.e., RTFO residues) as shown in Table 1, HMA mix additivities such as RAP, RAS, etc., tend to increase the proportion of the aged AB in the total AB blend [24,25,26]. Thus, in addition to their potential to increase the stiffness of the AB blend and ultimately that of the resulting HMA mix including rutting resistance improvements, these additives also have the possibility to impact the consistency/variability of the rheological properties of the plant-mix extracted ABs. As will be observed in the subsequent sections of this paper, whilst it was hypothesized that these additives and their dosage variations would significantly contribute to poor material consistency, some ABs with zero additives, in fact, exhibited more test data variability than those with RAP/RAS additives. This attests to the fact that it was more of the material-source effect that significantly contributed to the test repeatability and data variability than the type/dosage of the RAP/RAS additive per say.

### 3.4. Statistical Tools and Methods Used

For evaluating the data consistency, variability, and differences among the different AB sources/suppliers, the following statistical tools and methods were used in this study:

(1) Standard MS Excel descriptive statistics such as average (Avg) and CoV for assessing the data consistency, variability, and quality.
(2) Statistical analysis using *t*-tests, ANOVA, and Tukey’s HSD methods for assessing the differences among the different sources/suppliers in terms of the ABs’ rheological properties.

According to the AASHTO and ASTM test standards, a CoV threshold value of 3.20% (i.e., CoV ≤ 3.20%) for “single-operator precision” is recommended for the G*/Sin(δ) parameter based on the DSR testing of RTFO residue ABs [16,23]. In addition to the AASHTO and ASTM specification limits, a CoV threshold of 30% (i.e., CoV ≤ 30%) was also concurrently used in this study with the following sub-designations, as suggested in the literature [18,31,32,33]: (a) CoV ≤ 10% (excellent), (b) 10% < CoV ≤ 20% (good), (c) 20% < CoV ≤ 30% (marginal), and (d) CoV > 30% (poor). For the *t*-tests, ANOVA, and Tukey’s HSD methods, statistical analyses were performed at the typical 90, 95, and 99% confidence levels, respectively [18,34,35].

## 4. Laboratory Test Results and Analysis

The following section presents an analysis of the laboratory test results for 14 plant-mix extracted ABs (eight PG 64-22 and six PG 76-22), treated as RTFO residue, sourced from 14 different suppliers for the DSR test data that were measured at three temperatures, namely, 64, 70, and 76 °C, respectively. As previously mentioned, all the data used in this study were extracted from the DSS and do not include any detailed evaluation/analysis of the RAP/RAS effects [18,28].

### 4.1. The Asphalt-Binder DSR Test Results

Table 2 and Table 3 provide a list of the AB rheological properties, namely, the G*, δ, G*/Sin(δ), and G*/1 − (1/Tan(δ)Sin(δ)) parameters for PG64-22 at 64 and 70 °C temperatures, respectively, and 70 and 76 °C temperatures for PG 76-22, respectively. The test results represent a mean (Avg) value of three replicate specimens for each RTFO residue AB.

From Table 2, the G* parameter of PG 64-22 shows an average range of 5.27 kPa (Source04) to 8.46 kPa (Source07), with a CoV range of 4.09% to 57.87%, at 64 °C and 2.37 kPa (Source01) to 4.07 kPa (Source07), with a CoV range of 2.76% to 57.93%, at 70 °C. In general, Source07 exhibited the best performance in terms of the G* magnitude, while Source01 and Source04 were the poorest.

By comparison, Source09 (CoV > 30%) and Source06 (CoV > 25%) in Table 2 present more test data variability with the highest CoV values, while Source02 and Source04 exhibit the least test data variability with CoV values averaging 2.89%. However, with the exception of Source09, the CoV values are all less than 30%, indicating excellent (CoV ≤ 10%) to marginal (CoV ≤ 30%) data consistency [18,31,32,33]. Likewise, the δ parameter for PG 64-22 present an average range of 71.23° to 83.87° at 64 °C and 74.93° to 85.67 at 70 °C, respectively. As is evident in the table, variability in the δ data is very low with CoV values less than 5.0%—indicating high DSR test repeatability and excellent data consistency (CoV ≤ 10%) for this parameter [31,32,33].

From Table 3, the G* parameter of PG 76-22 shows an average range of 2.80 kPa (Source12) to 7.11 kPa (Source11), with a CoV range of 1.52% to 17.32% at 70 °C and 1.55 kPa (Source12) to 3.99 kPa (Source11), with a CoV range of 1.46% to 21.62% at 76 °C, respectively. By comparison, Source05 generally exhibited superior performance in terms of the G* magnitude while Source12 was the least. Source 05 seems to be an outlier and presented the highest G* value—over three times the other sources, registering 22.4 kPa at 64 °C and 9.64 kPa at 70 °C, respectively. The δ parameter, on the other hand, ranged from to 62.30° to 68.17° at 70 °C and 63.33° to 70.13° at 76 °C, respectively. Compared to PG 64-22, it is noted that, as theoretically expected, the PG 76-22 ABs, which are typically modified, exhibited higher G* and lower δvalues, respectively—indicating that they are much stiffer and more elastic at an equivalent temperature of 70 °C than the PG 64-22 ABs [14].

Although within the 30% threshold [18], Source13 presented more test data variability, with a CoV of 17.32%, while Source10 and Source14, which appear to represent the same supplier but probably sampled on different construction dates, exhibited the best test data consistency, with the least variability (CoV averaging 1.53%) [27,28,29]. Similarly, the δ parameter for Source10 and Source14 exhibited very low variability with CoV values, less than 1.00% [31,32,33]. By comparison, the δ parameter exhibited better consistency (i.e., lower CoV values) than the G* parameter.

### 4.2. Performance Ranking of the Asphalt-Binder Sources

Traditionally, the AB rheological property that has been used to correlate to rutting performance is the G*/Sin(δ) parameter—that is, the greater the G*/Sin(δ) in magnitude, the better the rutting resistance potential [8]. In 2001, the G*/(1 − (1/Tan(δ)Sin(δ))) parameter [21] was introduced to supplement the G*/Sin(δ) parameter for quantifying the high-temperature rutting susceptibility of ABs. Theoretically, Tan(δ) is assumed to capture the high-temperature rutting resistance of the ABs better than Sin(δ), as it tends to approach one at high temperatures [22]. Thus, the higher the G*/1 − (1/Tan(δ)Sin(δ) in magnitude, the better the rutting resistance potential for the AB. Based on these considerations, the rank order of superiority for the AB sources in terms of the magnitudes of the G*/Sin(δ) and G*/(1 − (1/Tan(δ)Sin(δ))) parameters are listed in Table 4.

According to Table 4a, the source with the best PG 64-22 AB in terms of potential for rutting resistance based on the G*/Sin(δ) and G*/(1 − (1/Tan(δ)Sin(δ))) magnitudes is Source07, followed by Source03, Source01 and Source04 present the worse ranking performance based on their lower G*/Sin(δ) and G*/(1 − (1/Tan(δ)Sin(δ))) values in Table 2. According to Table 1, both Source01 and Source04 had RAP/RAS additives. Therefore, their low ranking (Table 4a) could possibly be attributed to poor material quality including that of the RAP/RAS from these sources. This fact, may add poor-quality, and/or very old/aged RAP/RAS additives may not always automatically translate into significant stiffness enhancement, but the quality of the RAP/RAS material itself and the blending/mixing processes may also play a role. However, as noted previously, detailed evaluation of these effects was outside the scope of this paper. Further still, human and/or experimental errors could have probably contributed when extracting and testing the ABs for these particular sources.

In Table 4b, Source05 presents the best ranking performance for the PG 76-22 ABs—by over two times (Table 3) the second ranked Source11—while the worst ranking performer is Source12. Compared to Source12 without recycled materials, it is apparent that the presence of RAP and RAS in Source05 might have contributed to its superior ranking performance. This observation/finding suggests that material-source as well as the additives (i.e., RAP, RAS, etc.) could probably have had an impact on the high-temperature rheological properties of the ABs. Some sources, such as Source05, could be comprising highly superior/quality materials, including the RAP/RAS that significantly enhanced the AB’s rheological properties [25,26]. In general, Source05 and Source07 are the best sources, supplying superior performing ABs in terms of the high-temperature rheological properties and potential for rutting resistance.

Parametric-wise, both the G*/Sin(δ) and G*/(1 − (1/Tan(δ)Sin(δ))) parameters seemed to generally provide a similar ranking of the AB sources. For instance, both parameters are ranking Source07 and Source01 as the best and worst performers for PG 64-22, respectively. Similarly, Source01 and Source12 are ranked as the best and worst sources for PG 70-22, respectively.

### 4.3. The Actual Temperatures and PG Grades of the Asphalt-Binders

**Figure 1:**
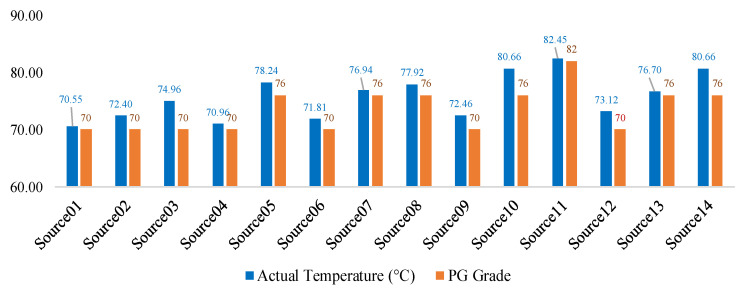
Actual and true temperature grades (G*/Sin(δ)).

The actual temperatures and PG grades are plotted in Figure 1. Note that for the purpose of this paper, original AB refers to neat AB before mixing and blending with aggregates and other additives such as RAP, RAS, lime, etc.—see Table 1. Based on the Superpave grading specification for RTFO residue ABs, the actual high-temperature grade is the temperature corresponding to a G*/Sin(δ) or G*/(1 − (1/Tan(δ)Sin(δ))) value of 2.2 kPa [16,18,28]. PG grade is a 6 °C point-step temperature grading system typically starting from 58 °C going upwards (i.e., PG 58-, PG 64-, PG 70-, PG 76-, PG 82-, etc.), with ABs usually grading downwards to the nearest temperature, e.g., 74.96 °C (actual) would grade as PG 70- as shown in Figure 1 [16].

For PG 64-22, the actual temperature and PG grades of the AB sources, as plotted in Figure 1, are higher than the temperature grades specified for the original AB in Table 1. This was theoretically expected considering that the DSR tests were conducted on short-term aged ABs that were extracted from plant-mix materials and treated as RTFO residue [18]. It is apparent from Figure 1 that the short-term aging along with the additives (i.e., RAP, RAS, etc.) stiffened up the PG 64-22 ABs and bumped the temperature grades upwards. Whilst majority of the sources jumped only by a one 6 °C step to PG 70-, Source07 and Source08 had a two 6 °C step jump to PG 76-, which may partially explain the superior performance and top ranking of Source07 in Table 2 and Table 4, respectively [18,30]. Evidently, these results demonstrate the sensitive nature of PG 64-22 ABs from these particular sources/suppliers to aging (short-term) and the impacts of RAP/RAS additives.

**Figure 2:**
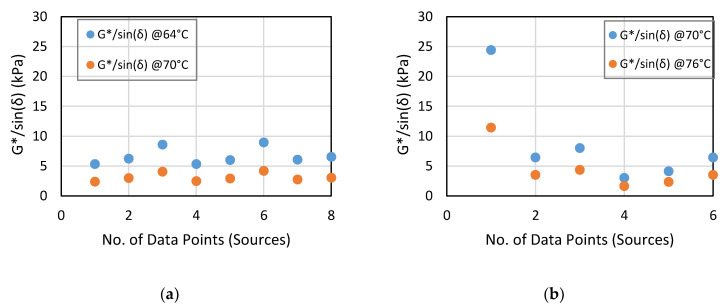
G*/Sin(δ) ranges for (**a**) PG 64-22 and (**b**) PG 76-22 rolling thin film oven (RTFO) residues.

By contrast, while the actual temperatures were generally higher (exceeding 76 °C), the PG grades of most of the PG 76-22 ABs in Figure 2 were, nonetheless, in conformity with the PG grades (PG 76-) of the original ABs specified in Table 1 [18]. In fact, only Source11 exhibited a one 6 °C grade bump to PG 82-, which partly explains its superior performance and high ranking in Table 4. From the perspective of the high-temperature rheological properties (i.e., G* and δ), these results suggest that the PG 76-22 ABs from these particular sources/suppliers were not very sensitive to short-term aging nor the impacts of RAP/RAS additives. However, detailed evaluation of the effects of the RAP/RAS effects, including chemical analysis, was outside the scope of this paper.

Comparing the different sources/suppliers, the actual temperature (at G*/Sin(δ) = 2.2 kPa) of Source07, which is 76.94 °C with a true PG grade of PG 76- in Figure 1, partly explains its superior performance and top ranking in Table 3 and Table 4, respectively. The same hypothesis could also explain Source12′s poor performance and poorest ranking amongst all the PG 76-22 ABs in Table 3 and Table 4, respectively. According to Figure 1, Source12 actually graded downwards by one 6 °C step to a PG 70-22 AB, with an actual measured temperature of 73.12 °C for G*/Sin(δ) = 2.2 kPa [16,18,27]. This is supported by the DSR test results in Table 3 that show a G*/Sin(δ) value of 1.65 kPa at 76 °C, which is 0.35 points less than the 2.0 kPa threshold [18,23], thus grading out as PG 70-, as shown in Figure 1. However, if the G*/(1 − (1/Tan(δ)Sin(δ))) ≥ 2.2 kPa parameter is considered, Source12 grades as a PG 76-22 AB. Therefore, the downward grading to PG 70- is considered to be related to the inadequacy of the G*/Sin(δ) parameter. Nonetheless, the overall results and findings suggest the need to be extra cautious with ABs sourced and supplied from Source12.

Looking at Table 3 (PG 76-22), the G*/(1 − (1/Tan(δ)Sin(δ))) parameter indicates the actual PG grade of all the sources is PG 76- or higher, which is not the case for the G*/Sin(δ) parameter. However, the G*/Sin(δ) and G*/(1 − (1/Tan(δ)Sin(δ))) values for the PG 64-22 RTFO residues in Table 2 are insignificantly different and all the sources grade out as PG 70- or higher for both parameters. As reported in the literature [21], this partially confirms the inadequacy of the G*/Sin(δ) parameter with respect to polymer modified ABs such as PG 76-22, which inadvertently alludes to the superiority of the G*/(1 − (1/Tan(δ)Sin(δ))) parameter.

For the particular sources and suppliers evaluated herein, the results in Figure 1 suggest that PG 64-22 is more sensitive to short-term aging and the impacts of RAP/RAS additives than PG 76-22 ABs. These findings evidently support the theoretical notion that PG 76-22, which is often polymer modified, has superior properties with better resistance to oxidative aging (short-term) than PG 64-22 ABs [18]. Based on the test results of Source12, it can also be concluded that one has to be cautious of the material-source effects and the use of the G*/Sin(δ) parameter for grading RTFO AB residues.

### 4.4. Test Data Consistency and Quality Ranking of the Asphalt-Binder Sources

The results in Table 2 and Table 3 represent an average of three replicates per source/supplier per AB type/grade, and were used for the statistical assessment of data variability through CoV analysis. Descriptive statistical analysis in terms of the CoV was used to comparatively evaluate the data quality and consistency in this study [31]. For the PG 64-22, only Source02 (@70 °C) and Source04 (@64 °C) satisfactorily met the AASHTO and ASTM CoV requirement of 3.20% for the G*/Sin(δ) parameter [16,25]. For PG 76-22, only Source10, Source11, and Source14 met the 3.20% CoV requirement for the G*/Sin(δ) parameter based on the AASHTO and ASTM standards [16,29].

Based on literature recommendations [18,27,31,32,33], a CoV threshold of 30% (i.e., CoV ≤ 30%) was also used as a supplementary measure of statistical variability—refer to Section 3.4 of this paper. With the exception of Source09 (CoV = 57.93%), the DSR test results in Table 2 and Table 3 exhibit reasonably acceptable repeatability and data consistency, with CoV values lower than 30% [18]. In addition to material-source quality, this good repeatability and relatively low variability in the test data, were partly attributed to good workmanship, proper machine calibration, the use of trained operators, etc. [18]. Quantitatively, the lower the CoV, the better the consistency and data quality.

On average, and based on the rankings in Table 2 and Table 3, Source01, Source02, Source03, Source04, Source10, Source11, Source12, and Source14 present the best sources in terms of test data consistency and possibly AB quality. These sources are associated with the lowest CoV values (Table 2 and Table 3) and are top ranked (1st to 3rd positions) in Table 5—suggesting good quality-control practices. In particular, Source10 and Source14, from the same plant/supplier but sampled on different construction dates, indicate the same consistency in AB rheological properties (Table 3) over time and had the same top ranking (1st) in Table 5; this may suggest good quality-control (QC) practices with this supplier.

Source05, Source06, Source07, Source09, and Source13, on the other hand, present relatively high CoV values (although lower than 30% except for Source09)—see Table 2 and Table 3 [18,31,32,33]. Considering that the DSR tests were conducted in the same laboratory and on the same equipment, the test data variability associated with these sources could be partially attributed to inadequate quality control practices from the source/supplier as well as the possibilities of human test errors. Thus, solely based on material-source quality and test data consistency, these six sources would not be among the top preferences for supplying the ABs.

Of interest in Table 2 and Table 3 is also the observation that some ABs without RAP/RAS additives (e.g., Source08, Source12, Source13, etc.) exhibited more test data variability than most of those with RAP/RAS additives such as Source04 or Source10. In fact, all the top-ranked (1st and 2nd) sources with the best test data consistency (i.e., lowest CoV values) for both PG 64-22 and PG 76-22 ABs in Table 5 comprise RAP/RAS additives. By inference and as previously mentioned in Section 3.3, these findings attest to the fact that it was more of the material-source effect that significantly contributed to the test repeatability, consistency, and data variability than the type and/or dosage of the RAP/RAS additives—that is, the material-source effect had a more profound effect on the DSR test data variability than the type/dosage of the RAP/RAS additive. However, detailed evaluation of this aspect was outside the scope of this paper.

Overall, the ranking results in Table 2 and Table 3 imply that consistency and quality aspects, just like performance superiority (and costs of course), are critical issues in deciding the AB source and supplier. Thus, the material-source effects should be holistically studied and evaluated from both the response-behavior (rheological properties) and quality (consistence) standpoints as well as the economical aspects.

## 5. Statistical Analyses and Material-Source Effects

To further assess the variability associated with the AB sources/suppliers and ascertain if the sources were statistically significantly different, ANOVA was performed with respect to the rheological properties, namely, the G*, δ, G*/Sin(δ), and G*/1 − (1/Tan(δ)Sin(δ)) parameters. The ANOVA analysis was performed using open-source statistical software R [36]. The ANOVA statistical results, based on three replicates per AB source per test temperature, are presented in Table 5 at the standard 95% confidence level (CL) (i.e., α = 5.0% = 0.05) in terms of the *p*-values. Interpretively, if *p*-value is less than α, i.e., *p*-value < 0.05, then there is some potentially statistical differences among the AB sources/suppliers with respect to that particular parameter and vice versa [35].

From Table 5, the ANOVA analysis shows that the probability value (*p*-value) was lower than 5.0% for all the ABs and temperatures with respect to the δ and G*/1 − (1/Tan(δ)Sin(δ)) parameters—meaning that, at a 95% confidence level (CL), there is at least one source/supplier that is statistically different from the other sources. On the other hand, the G* and G*/Sin(δ) parameters indicate a statistically significant difference for the PG 76-22 AB while suggesting no statistical difference among the PG 64-22 ABs for the two temperatures indicated in Table 5. With respect to material-source, it can also be inferred that the phase angle (δ) has a more profound influence on the ANOVA results with respect to the G*/1 − (1/Tan(δ)Sin(δ)) parameter. Similar statistical results and findings were observed at 90 and 99% CLs, respectively, and hence it was deemed an unnecessary duplication to present them in this paper.

Although the ANOVA analysis provides a first insight into the statistical differences among the AB sources, a Tukey Post Hoc Test (HSD) at a 95% CL was also conducted to determine which pairs of the sources had significant differences among the rheological properties [34]. This test essentially compares if the differences in the means (Avg) of each source-pair are greater than the expected standard error from all the other pairs for the PG 76-22 AB sources. One advantage of the Tukey Post Hoc Test over ANOVA analysis is that it greatly reduces Type I errors (i.e., rejection of a true null hypothesis) [17]. A “True-False” methodology was proposed to denote that the differences in the rheological properties under evaluation between each pair of the AB source were high enough to be considered statistically different. The True-False results of the Tukey Post Hoc Test are listed in Table 6. In simple interpretive terms, “True” in Table 6 means that the paired sources are statistically significantly different, whereas “False” means that the paired sources are statistically indifferent (i.e., similar).

Table 6 confirms the results from ANOVA analysis that the G* and G*/Sin(δ) parameters indicate no major significant differences for the PG 64-22 AB sources but shows some differences with respect to the PG 76-22 AB sources. When analyzing which rheological parameter is the most statistically different among the AB sources, it was found that the δ parameter presents the most statistical difference and sensitivity (i.e., “True” response in Table 6) for both PG 64-22 and PG 76-22 AB sources, followed by the G*/1 − (1/Tan(δ)Sin(δ)) parameter. The G* and G*/Sin(δ) parameter exhibited the least statistical differences among the sources with a lot of “False” responses and very few “True” responses. Additionally, the sources for PG 64-22 ABs exhibited comparatively few differences (i.e., fewer “True” and more “False” responses) than the PG 76-22 AB sources.

The most different sources (i.e., “True” responses) for PG 64-22 ABs at both 64 and 70 °C test temperatures were Source04–Source07 and Source01–Source07, respectively, with 50% of the statistical runs being significantly different. For PG 76-22 ABs, Source 05 exhibited significant differences from all the other sources at both 70 and 76 °C test temperatures. By contrast, the source-pairs that are statistically indifferent and present similar average values with a “False” response for most of the parameters in Table 6 are Source03–Source06, Source03–Source09, Source04–Source08, Source06–Source09, Source08–Source09, and Source10–Source14 (same plant/supplier), respectively. Based on Table 6 and the “False” responses indicated, these sources did not indicate any rheological parameters that could be considered statistically different.

Overall, Table 6 suggests that δ is the most sensitive parameter to material-source effects followed by the G*/1 − (1/Tan(δ)Sin(δ)) parameter. The G* and G*/Sin(δ) parameters, on the other hand, indicated the least statistical sensitivity to the material-source effect. On this basis, this study suggests using the δ and G*/1 − (1/Tan(δ)Sin(δ)) parameters for evaluating the effects of material sources and suppliers on ABs extracted from plant-mix materials and treated as RTFO residue.

## 6. Synthesis and Discussions of the Test Results

To further assess the statiscal spreads associated with the AB sources in terms of the measured rheological properties and statistical variability (CoV), the range and mean (Avg) values for these parameters were determined and are summarized in Table 7 and Table 8.

**Figure 3:**
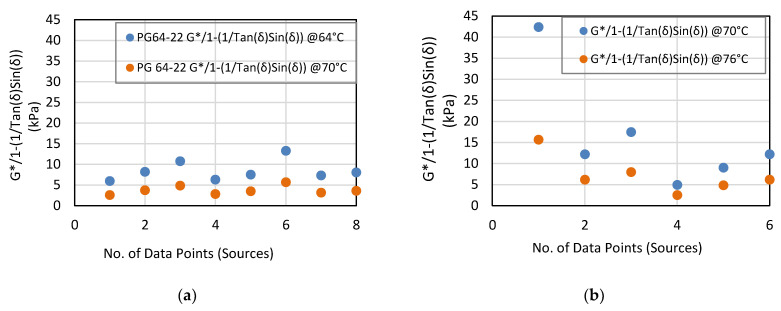
G*/1 − (1/Tan(δ)Sin(δ)) ranges for (**a**) PG 64-22 and (**b**) PG 76-22 RTFO residues.

From Table 7, the values of the G*, G*/Sin(δ) and G*/(1 − (1/Tan(δ)Sin(δ))) parameters for PG 64-22 AB are all lower than PG 76-22 and vice versa for the δ parameter, as theoretically expected [37,38,39]. For each AB type/grade, a similar trend was also evident with respect to temperature effects—The values are higher at the lower temperature and vice versa for the δ parameter. The graphical plot in Figure 2 shows that the G*/Sin(δ) for PG 64-22 and PG 76-22 ranges from 1.0 to 10 kPa and 1.0 to 25 kPa, respectively—while Figure 3 shows a range of 2.0 to 15 kPa and 2.0 to 45.0 kPa for the G*/1 − (1/Tan(δ)Sin(δ)) parameter, respectively. However, if Source05 (data point #1 in Figure 2b and Figure 3b) is assumed to be an outlier, the ranges for PG 76-22 would be 1.0 to 10 kPa and 2.0 to 20.0 kPa, respectively. Furthermore, unlike the G*/Sin(δ)) parameter in Figure 2 and Figure 3, the G*/(1 − (1/Tan(δ)Sin(δ))) parameter shows that all the AB sources met the 2.2 kPa minimum value [21].

**Figure 4:**
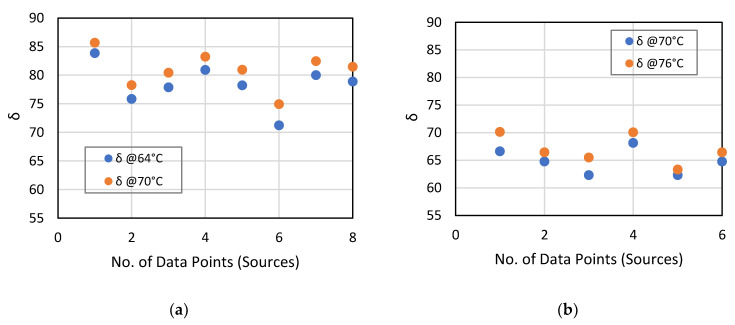
δ ranges for (**a**) PG 64-22 and (**b**) PG 76-22 RTFO residues.

With respect to the phase angle, Figure 4 shows a range of 70 to 90 °C (more viscous) for PG 64-22 and 60 to 80 °C (more elastic) for PG 76-22, respectively. Coincidentally, all the sources satisfactorily meet the 55° thresholds with all the δ values being greater than 60°.

In terms of the test data variability, the δ parameter presented the smallest and least range of CoV values—indicating the best data consistency. In fact, the overall CoV range for the δ parameter in Table 8 is 0.05 to 1.50%, with an average of 0.97%. The rest of the other parameters, being computed as function of G* and δ, exhibited an almost similar CoV range of 1.00% to 60.00%, with the G*/1 − (1/Tan(δ)Sin(δ) parameter exhibiting the highest variability followed by G*/Sin(δ). In terms of AB comparisons, PG 64-22 surprisingly exhibited the higher statistical variability with a CoV range of 1.00% to 60% versus 0.05% to 25.00% for PG 76-22. As previously discussed in Section 4, this was partly due to one outlier, namely, Source09, which was associated with comparatively very high variability in its DSR test data—see Table 2.

For the ABs (RTFO residue) evaluated, the PG 64-22 ABs, consistent with theoretical expectations, exhibited lower stiffness values as quantified in terms of the rheological properties (G*, G*/Sin(δ)) and G*/1 − (1/Tan(δ)Sin(δ)) than the PG 76-22 ABs at equivalent temperatures—and vice versa for the δ parameter [39].

In addition, it is important to note that, of all the sources evaluated in this study, only one source (Source09) exhibited unacceptably high variability in the DSR test data. Without discounting other probable contributing factors, such as good workmanship during laboratory DSR testing or effects of additives such as RAP and RAS, this observation suggests that the Texas AB suppliers (over 90%) are, in general, adhering to good quality-control (QC) practices in their production processes.

Finally, ANOVA analysis showed that at least one source was statistically significantly different from the other sources with respect to the δ and G*/1 − (1/Tan(δ)Sin(δ)) parameters. On the other hand, a Tukey’s HSD analysis indicated large variation among the PG 76-22 AB sources, whilst the sources for PG 64-22 ABs exhibited comparatively few differences. Parametric wise, the δ parameter presented the most statistical difference and sensitivity for both PG 64-22 and PG 76-22 AB sources (followed by G*/1 − (1/Tan(δ)Sin(δ))), whilst the G* and G*/Sin(δ) parameters exhibited the least statistical differences among the AB sources.

## 7. Conclusions and Recommendations

The key findings, conclusions, and recommendations drawn from the study are summarized below.

(1) Asphalt-binders that are classified under a particular PG grade can exhibit different physical and rheological properties.
(2) The study findings indicate that the δ and G*/(1 − (1/Tan(δ)Sin(δ))) were the best parameters for assessing and quantifying the sensitivity of the material-source effects on the high-temperature rheological properties of RTFO residue ABs (namely, plant-mix recovered ABs). The G*/(1 − (1/Tan(δ)Sin(δ))) parameter exhibited superiority over the G*/Sin(δ) parameter in detecting and statistically quantifying the ABs’ high-temperature sensitivity to material-source effects.
(3) Based on the true PG high-temperature grades of the 14 sources of ABs, the test results suggest that PG 64-22 is more sensitive to short-term aging and the impacts of RAP/RAS additives than PG 76-22 ABs. These findings evidently support the fundamental theory that PG 76-22, which is often polymer modified, has superior rheological properties with better high-temperature tolerance and resistance to oxidative aging (short-term) than PG 64-22 ABs.
(4) The study highlights the sensitive nature of the high-temperature rheological properties of ABs (i.e., RTFO residues) extracted from plant-mix materials to material-source effects, including the potential impacts on the PG grading and high-temperature performance (e.g., stiffness, rutting potential, etc.) of the Abs.
(5) To further supplement the results and findings presented in this paper, future follow-up studies should cover more sources/suppliers and AB types/grades (e.g., PG 70–22), including detailed evaluations of the RAP/RAS effects that were outside the scope of this study.

## Figures and Tables

**Table 1:** Plant-mix extracted asphalt-binders.

Source	DSS Section ID	Asphalt-Binder	Corresponding HMA Mix Additives
Source01	TxDOT_TTI-00001	PG 64-22	Type D = Quartzite + 20.10% RAP (10.20% coarse + 9.90% fine)
Source02	TxDOT_TTI-00004	PG 64-22	Type B = Limestone/dolomite + 29.90% RAP + 1.00% hydrated lime
Source03	TxDOT_TTI-00005	PG 64-22	Type C = Limestone/dolomite/gravel + 20.10% RAP + 1% lime
Source04	TxDOT_TTI-00015	PG 64-22	Type C = Limestone/dolomite + 17.00% RAP + 3.00% RAS
Source06	TxDOT_TTI-00040	PG 64-22	Type D = 17.10% RAP + 2.60% RAS
Source07	TxDOT_TTI-00032	PG 64-22	Type D = 15.00% RAP + 4.20% RAS
Source08	TxDOT_TTI-00042	PG 64-22	Type C = Limestone/dolomite
Source09	TxDOT_TTI-00028	PG 64-22	Type D = Sandstone/limestone/dolomite + 11.90% RAP
Source05	TxDOT_TTI-00009	PG 76-22	Type B = Limestone/dolomite/gravel + 21.90% RAP + 3.00% RAS
Source10	TxDOT_TTI-00004	PG 76-22	SMA = Limestone/dolomite + 12.10% RAP + 1.00% hydrated lime
Source11	TxDOT_TTI-00006	PG 76-22	PFC = Sandstone/limestone/dolomite + 1.00% hydrated lime
Source12	TxDOT_TTI-00007	PG 76-22	Type F =Sandstone + 1% hydrated lime
Source13	TxDOT_TTI-00007	PG 76-22	PFC = Sandstone + 1% hydrated lime
Source14	TxDOT_TTI-00046	PG 76-22	SMA = Limestone/dolomite + 12.10% RAP + 1.00% hydrated lime

**Table 2:** Dynamic shear rheometer (DSR) test results at 64 and 70 °C for PG 64-22 asphalt binder.

RTFO Residue		Temperature = 64 °C				Temperature = 70 °C			
Source	Asphalt-Binder Type/Grade	G* (kPa) (CoV)	δ (CoV)	G*/Sin(δ) (CoV)	G*/1 − (1/Tan(δ)Sin(δ)) (CoV)	G* (kPa) (CoV)	δ (CoV)	G*/Sin(δ) (CoV)	G*/1 − (1/Tan(δ)Sin(δ)) (CoV)
Source01	PG 64-22	5.30	83.87	5.33	5.94	2.37	85.67	2.38	2.56
		(5.31%)	(0.17%)	(5.43%) [4] *	(5.59%) [2]	(4.06%)	(0.18%)	(4.04%) [2]	(4.36%) [2]
Source02	PG 64-22	6.05	75.87	6.24	8.17	2.93	78.27	2.99	3.71
		(4.09%)	(0.15%)	(4.21%) [2]	(4.39%) [1]	(2.76%)	(0.15%)	(2.71%) [1]	(3.02%) [1]
Source03	PG 64-22	8.39	77.90	8.58	10.75	4.02	80.43	4.08	4.85
		(4.96%)	(0.22%)	(5.02%) [3]	(5.33%) [3]	(4.10%)	(0.26%)	(4.17%) [3]	(4.47%) [3]
Source04	PG 64-22	5.27	80.93	5.34	6.29	2.48	83.23	2.50	2.82
		(3.11%)	(0.38%)	(3.17%) [1]	(3.74%) [4]	(4.45%)	(0.37%)	(4.42%) [4]	(5.09%) [4]
Source06	PG 64-22	5.87	78.23	6.00	7.48	2.91	80.97	2.94	3.47
		(25.92%)	(1.03%)	(26.16%) [7]	(28.08%) [7]	(23.86%)	(0.82%)	(24.27%) [4]	(25.44%) [4]
Source07	PG 64-22	8.46	71.23	8.96	13.26	4.07	74.93	4.21	5.66
		(15.14%)	(1.30%)	(15.89%) [6]	(18.65%) [5]	(15.22%)	(1.32%)	(15.59%) [5]	(18.31%) [6]
Source08	PG 64-22	5.99	80.03	6.09	7.30	2.74	82.47	2.76	3.16
		(15.36%)	(0.47%)	(15.46%) [5]	(16.27%) [6]	(16.47%)	(0.28%)	(16.54%) [6]	(16.98%) [5]
Source09	PG 64-22	6.42	78.90	6.54	8.04	3.02	81.50	3.05	3.57
		(57.87%)	(0.34%)	(57.87%) [8]	(58.57%) [8]	(57.93%)	(0.37%)	(58.05%) [8]	(58.55%) [8]

Legend: PG = Performance-graded; G* = Complex shear modulus; δ = Phase angle; CoV = Coefficient of variation; Tan(δ) = Tangent of the phase angle; Sin(δ) = Sine of the phase angle; * [i] = Ranking based on CoV results.

**Table 3:** PG 76-22 DSR test results at 70 and 76 °C.

RTFO Residue		Temperature = 70 °C				Temperature = 76 °C			
Source	Asphalt-Binder Type/Grade	G* (kPa) (CoV)	δ (CoV)	G*/Sin(δ) (CoV)	G*/1 − (1/Tan(δ)Sin(δ)) (CoV)	G* (kPa) (CoV)	δ (CoV)	G*/Sin(δ) (CoV)	G*/1 − (1/Tan(δ)Sin(δ)) (CoV)
Source05	PG 76-22	22.40	66.63	24.43	42.38	9.64	70.13	11.43	15.68
		(11.40%)	(0.53%)	(11.70%) [4] *	(13.05%) [4]	(21.62%)	(0.59%)	(12.98%) [4]	(22.47%) [5]
Source10	PG 76-22	5.83	64.77	6.46	12.20	3.25	66.46	3.55	6.18
		(1.53%)	(0.09%)	(1.55%) [1]	(1.85%) [1]	(1.46%)	(0.17%)	(1.50%) [1]	(2.02%) [1]
Source11	PG 76-22	7.11	62.30	8.03	17.46	3.99	65.50	4.39	7.99
		(3.31%)	(0.16%)	(3.38%) [2]	(3.80%) [2]	(2.28%)	(0.31%)	(2.28%) [2]	(2.66%) [2]
Source12	PG 76-22	2.80	6.,17	3,01	4,93	1.55	70.07	1.65	2.53
		(7.51%)	(0.86%)	(7.97%) [3]	(10.05%) [3]	(8.08%)	(0.95%)	(8.20%) [3]	(10.60%) [3]
Source13	PG 76-22	3.66	62.30	4.13	9.01	2.11	63.33	2.36	4.84
		(17.32%)	(0.42%)	(17.39%) [5]	(19.34%) [5]	(16.29%)	(0.46%)	(16.80%) [5]	(18.34%) [4]
Source14	PG 76-22	5.85	64.77	6.47	12.21	3.24	66.47	3.54	6.18
		(1.52%)	(0.09%)	(1.55%) [1]	(1.85%) [1]	(1.46%)	(0.17%)	(1.49%) [1]	(2.02%) [1]

Legend: PG = Performance-graded; G* = Complex shear modulus; δ = Phase angle; CoV = Coefficient of variation, Tan(δ) = Tangent of the phase angle; Sin(δ) = Sine of the phase angle; * [i] = Ranking based on CoV results.

**Table 4:** Ranking of the asphalt-binders based on the rheological properties.

**(a) PG 64-22 (RTFO Residue)**		**Temperature = 64 °C**		**Temperature = 70 °C**	
**Source**	**Asphalt-Binder Type/Grade**	G*/Sin(δ)	G*/1 − (1/Tan(δ)Sin(δ))	G*/Sin(δ)	G*/1 − (1/Tan(δ)Sin(δ))
Source01	PG 64-22	8	8	8	8
Source02	PG 64-22	4	3	4	3
Source03	PG 64-22	2	2	2	2
Source04	PG 64-22	7	7	7	7
Source06	PG 64-22	6	5	5	5
Source07	PG 64-22	1	1	1	1
Source08	PG 64-22	5	6	6	6
Source09	PG 64-22	3	4	3	4
**(b) PG 76-22 (RTFO Residue)**		**Temperature = 70 °C**		**Temperature = 76 °C**	
**Source**	**Asphalt-Binder Type/Grade**	G*/Sin(δ)	G*/1 − (1/Tan(δ)Sin(δ))	G*/Sin(δ)	G*/1 − (1/Tan(δ)Sin(δ))
Source05	PG 76-22	1	1	1	1
Source10	PG 76-22	3	3	3	3
Source11	PG 76-22	2	2	2	2
Source12	PG 76-22	5	5	5	5
Source13	PG 76-22	4	4	4	4
Source14	PG 76-22	3	3	3	3

Legend: PG = Performance-graded; G* = Complex shear modulus; δ = Phase angle; Tan(δ) = Tangent of the phase angle; Sin(δ) = Sine of the phase angle.

**Table 5:** ANOVA statistical results (*p*-values).

ANOVA *p*-Value (Three Replicates Per Asphalt-Binder Source Per Test Temperature)				
Parameter	PG 64-22 @64 °C	PG 64-22 @70 °C	PG 76-22 @70 °C	PG 76-22 @76 °C
G* (kPa)	0.113	0.0717	4.12 × 10^−10^	1.09 × 10^−6^
δ	6.05 × 10^−14^	2.76 × 10^−13^	8.28 × 10^−11^	1.18 × 10^−10^
G*/Sin(δ)	0.0793	0.0575	5.91 × 10^−10^	3.36 × 10^−9^
G*/1 − (1/Tan(δ)Sin(δ))	0.00891	0.011	3.40 × 10^−9^	3.52 × 10^−6^

Legend: PG = Performance-graded; G* = Complex shear modulus; δ = Phase angle; CoV = Coefficient of variation; Tan(δ) = Tangent of the phase angle; Sin(δ) = Sine of the phase angle, @ = at a temperature of °C.

**Table 6:** Tukey HSD statistical results (True-False) @ 95% confidence level (CL).

**Source-Pair**	**PG 64-22 @64 °C (RTFO Residue)**				**PG 64-22 @70 °C (RTFO Residue)**			
	G* (kPa)	**δ**	G*/Sin(δ)	G*/1 − (1/Tan(δ)Sin(δ))	G* (kPa)	**δ**	G*/Sin(δ)	G*/1 − (1/Tan(δ)Sin(δ))
S2 ≠ S1	False	True	False	False	False	True	False	False
S3 ≠ S1	False	True	False	False	False	True	False	False
S4 ≠ S1	False	True	False	False	False	True	False	False
S6 ≠ S1	False	True	False	False	False	True	False	False
S7 ≠ S1	False	True	False	True	False	True	False	True
S8 ≠ S1	False	True	False	False	False	True	False	False
S9 ≠ S1	False	True	False	False	False	True	False	False
S3 ≠ S2	False	True	False	False	False	True	False	False
S4 ≠ S2	False	True	False	False	False	True	False	False
S6 ≠ S2	False	True	False	False	False	True	False	False
S7 ≠ S2	False	True	False	False	False	True	False	False
S8 ≠ S2	False	True	False	False	False	True	False	False
S9 ≠ S2	False	True	False	False	False	True	False	False
S4 ≠ S3	False	True	False	False	False	True	False	False
S6 ≠ S3	False	False	False	False	False	False	False	False
S7 ≠ S3	False	True	False	False	False	True	False	False
S8 ≠ S3	False	True	False	False	False	True	False	False
S9 ≠ S3	False	False	False	False	False	False	False	False
S6 ≠ S4	False	True	False	False	False	True	False	False
S7 ≠ S4	False	True	False	True	False	True	False	True
S8 ≠ S4	False	False	False	False	False	False	False	False
S9 ≠ S4	False	True	False	False	False	True	False	False
S7 ≠ S6	False	True	False	False	False	True	False	False
S8 ≠ S6	False	True	False	False	False	True	False	False
S9 ≠ S6	False	False	False	False	False	False	False	False
S8 ≠ S7	False	True	False	True	False	True	False	False
S9 ≠ S7	False	True	False	False	False	True	False	False
S9 ≠ S8	False	False	False	False	False	False	False	False
**Source-Pair**	**PG 76-22 @70 °C (RTFO Residue)**				**PG 76-22 @76 °C (RTFO Residue)**			
	G* (kPa)	**δ**	G*/Sin(δ)	G*/1 − (1/Tan(δ)Sin(δ))	G* (kPa)	**δ**	G*/Sin(δ)	G*/1 − (1/Tan(δ)Sin(δ))
S11 ≠ S10	False	True	False	False	False	False	False	False
S12 ≠ S10	True	True	True	True	False	True	True	False
S13 ≠ S10	False	True	False	False	False	True	False	False
S14 ≠ S10	False	False	False	False	False	False	False	False
S5 ≠ S10	True	True	True	True	True	True	True	True
S12 ≠ S11	True	True	True	True	True	True	True	True
S13 ≠ 11	True	False	True	True	False	True	True	False
S14 ≠ S11	False	True	False	False	False	False	False	False
S5 ≠ S11	True	True	True	True	True	True	True	True
S13 ≠ S12	False	True	False	False	False	True	False	False
S14 ≠ S12	True	True	True	True	False	True	True	False
S5 ≠ S12	True	True	True	True	True	False	True	True
S14 ≠ S13	False	True	False	False	False	True	False	False
S5 ≠ S13	True	True	True	True	True	True	True	True
S5 ≠ S14	True	True	True	True	True	True	True	True

Legend: PG= Performance-graded; G* = Complex shear modulus; δ = Phase angle; CoV = Coefficient of variation; Tan(δ) = Tangent of the phase angle; Sin(δ)= Sine of the phase angle; S# = Source(##), i.e., S11 ≠ S10 = Source11 not similar to Source10; True = the paired sources are statistically significantly different; False = the paired sources are statistically indifferent (i.e., similar).

**Table 7:** Measured range of the asphalt-binder rheological parameters.

Asphalt-Binder	Temperature (°C)	Measured Parametric Range (RTFO Residue)			
		G* (kPa)	Δ (°)	G*/Sin(δ) (kPa)	G*/1 − (1/Tan(δ)Sin(δ)) (kPa)
PG 64-22	64	5.27–8.46	71.23–83.87	5.33–8.96	5.94–13.26
		(3.19)	(12.64)	(3.63)	(7.32)
	70	2.37–4.07	74.93–85.67	2.38–4.21	2.56–5.66
		(1.70)	(10.74)	(1.83)	(3.10)
PG 76-22	70	2.80–22.4	62.30–68.17	3.01–24.43	4.93–42.38
		(19.60)	(5.87)	(21.42)	(37.45)
	76	1.55–9.64	63.33–70.13	1.65–11.43	2.53–15.68
		(8.09)	(6.80)	(9.78)	(13.15)

Legend: PG = Performance-graded; G* = Complex shear modulus; δ = Phase angle; CoV = Coefficient of variation; Tan(δ) = Tangent of the phase angle; Sin(δ) = Sine of the phase angle.

**Table 8:** Statistical variability (coefficient of variation (CoV) ranges) of the rheological parameters.

Asphalt-Binder	Temperature (°C)	Computed CoV Range (RTFO Residue)			
		G* (kPa) (*)	δ (°) (*)	G*/Sin(δ) (*)	G*/1 − (1/Tan(δ)Sin(δ)) (*)
PG 64-22	64	3.11–57.87%	0.15–1.30%	3.17–57.87%	3.74–58.57%
		(54.76%)	(1.15%)	(54.70%)	(54.83%)
	70	2.76–57.93%	0.15–1.32%	2.71–58.05%	3.02–58.55%
		(55.17%)	(1.17%)	(55.34%)	(55.53%)
PG 76-22	70	1.52–17.32%	0.09–0.86%	1.55–17.39%	1.85–19.34%
		(15.80%)	(0.77%)	(15.84%)	(17.49%)
	76	1.46–21.62%	0.17–0.95%	1.49–16.80%	2.02–22.47%
		(20.16%)	(0.78%)	(15.31%)	(20.45%)

Legend: PG = Performance -graded; G* = Complex shear modulus; δ = Phase angle; CoV = Coefficient of variation; Tan(δ) = Tangent of the phase angle; Sin(δ) = Sine of the phase angle; *Average.

## Data Availability

Data are available upon request to the corresponding author.

## List of Abbreviations and Symbols

AB	Asphalt-Binder
AASHTO	American Association of State Highway and Transportation Officials
ASTM	American Society for Testing and Materials
CoV	Coefficient of Variation, %
DSR	Dynamic Shear Rheometer
DSS	Texas Data Storage System
G*/sinδ	Complex Modulus G* Divided by the Sine of the Phase Angle δ
HMA	Hot-Mix Asphalt
Hwy	Highway
PG	Performance Grade
QC/QA	Quality Control/Assurance
RAP	Recycled Asphalt Pavement
RAS	Recycled Asphalt Shingles
SMA	Stone Mastic Asphalt
PFC	Permeable Friction Course
Avg	Average
@	At a Temperature of __ °C
TxDOT	Texas Department of Transportation
TTI	Texas A&M Transportation Institute
